# BNIP3 decreases the LPS-induced inflammation and apoptosis of chondrocytes by promoting the development of autophagy

**DOI:** 10.1186/s13018-020-01791-7

**Published:** 2020-07-28

**Authors:** Zetao Ma, Deli Wang, Jian Weng, Sheng Zhang, Yuanshi Zhang

**Affiliations:** Orthopaedic Department, PKU Shenzhen Hospital, No. 1120, Road Lianhua, Shenzhen, 518036 Guangdong Province China

**Keywords:** Autophagy, BNIP3, Chondrocytes, Inflammation, Apoptosis

## Abstract

**Background:**

Inflammation and apoptosis of chondrocytes are the pathological bases of osteoarthritis. Autophagy could alleviate the symptoms of inflammation and apoptosis. Previous study has shown that BCL2/adenovirus E1B 19 kDa protein-interacting protein 3 (BNIP3) can induce the occurrence and development of autophagy. However, it is unknown whether autophagy induced by BNIP3 can alleviate the inflammation and apoptosis of chondrocytes.

**Methods:**

We used the lentivirus to construct the overexpression BNIP3 chondrocytes. Next, the lipopolysaccharide (LPS) was used to stimulate these cells to simulate the physiological environment of osteoarthritis. After that, the enzyme-linked immunosorbent assays (ELISA) were performed to determine the levels of tumor necrosis factor-α (TNF-α), interleukin-1 beta (IL-1β), and interleukin-6 (IL-6) and the flow cytometry was performed to detect the apoptosis rates of chondrocytes. At last, the expression of autophagy-related proteins was detected with the western blotting.

**Results:**

The expression of BNIP3 was suppressed after treatment with LPS. However, overexpression of BNIP3 inhibited the secretion of proinflammatory factors (TNF-α, IL-1β, and IL-6) and decreased the apoptosis of chondrocytes. Furthermore, overexpression of BNIP3 led to the upregulation of autophagy-related protein expression including little computer 3 (LC3), autophagy-related protein 7 (ATG7), and Beclin-1. Application of autophagy inhibitor recovered the expression of proinflammatory factors and apoptosis rates of chondrocytes.

**Conclusions:**

BNIP3 decreased the LPS-induced inflammation and apoptosis of chondrocytes by activating the autophagy.

## Background

Bone destruction and osteophyte formation are the major features of osteoarthritis and the main clinical symptom of osteoarthritis is severe joint pain [1]. Chondrocytes are the most common cells in articular cartilage and play a critical role in the proliferation and degradation of chondrocytes [2]. However, the study also indicated that chondrocyte degradation induced by chondrocyte inflammation was associated with the occurrence and development of osteoarthritis [3]. The development and progression of osteoarthritis which affected about 10-15% of adults worldwide could restrict the mobility and eventually lead to the disability of patients [4]. Therefore, osteoarthritis is a global public health problem, which affects the living standards of patients. And we need to clarify the molecular mechanisms of the occurrence of osteoarthritis to develop targeted therapies.

Autophagy is a normal physiological function and protective mechanism of the human body. Autophagy is a degradation process in the cell, which is initiated under extreme conditions such as hypoxia and low glucose to maintain intracellular homeostasis [5]. Autophagy could be divided into large autophagy, micro-autophagy, and mediated autophagy according to the diverse modes of the interaction between degraded substance and lysosome [6]. There is study has shown that autophagy can protect the body against the external environment by activating immune responses (antigen presentation, cytokine secretion, and production of antimicrobial peptides) [7]. Furthermore, the autophagy process was associated with the occurrence and development of inflammation. The study showed that the restriction of autophagy led to the aggravation of intestinal inflammation [8, 9]. In addition, there is research suggesting that the autophagy alleviates high glucose-induced inflammation and damage of podocyte and thus relieves the symptoms of diabetic nephropathy [10]. And all these results indicated that the autophagy process could protect multiple types of organs from the inflammatory injury.

BCL2/adenovirus E1B 19 kDa protein-interacting protein 3 (BNIP3) was one kind of the Bcl2 and adenovirus E1B 19 kDa-interacting proteins [11]. BNlP3 is expressed in almost all animal and human tissue cells and is strongly positive in various human tumor cells [12]. BNIP3 protein is also a part of the transmembrane protein and forms specific protein dimers with various types of anti-apoptotic proteins by its unique BH3 structure outside the membrane to promote cell apoptosis. There was a TM region in the mitochondrial membrane so that BNIP3 was anchored in the mitochondria to promote apoptosis, while autophagy was suppressed in the absence of BNIP3 [13]. BNIP3 could induce the mitochondrial dysfunction and lead to cell death through apoptosis and autophagy [14–17]. Furthermore, study indicated that the BNIP3 combined with ceramide could induce the autophagy and lead to the death of glioma cells [18]. And the research suggested that BNIP3-induced autophagy in retinal pigment epithelial cells could reduce the expression of interleukin-18 (IL-18) and the proportion of apoptotic cells [19]. However, whether BNIP3 could decrease inflammation damage of chondrocyte by inducing autophagy is unclear.

In this study, we establish the overexpression BNIP3 chondrocytes and use the lipopolysaccharide (LPS) to stimulate these cells to simulate the physiological environment of osteoarthritis. Next, the enzyme-linked immunosorbent assay (ELISA) and flow cytometry were performed to clarify the levels of inflammation factors and proportion of apoptosis cells. At last, the expression of autophagy-related proteins was determined with the western blotting. According to the results of those experiments, we could illuminate the effect of BNIP3-induced autophagy on the inflammation and apoptosis of chondrocytes.

## Material and methods

### Cell culture and transfection

Chondrogenic cell line ATDC5 from mouse was obtained from Sigma-Aldrich (St. Louis, MO, USA). These cells were cultured with the medium which was the mixture (1:1) of Dulbecco’s Modified Eagle Medium (DMEM) and Ham’s F12. And the mixture medium was supplemented with 5% fetal bovine serum (Gibco, Thermo Fisher Scientific, USA) and 2 mM Glutamine (Sigma-Aldrich). These cells were placed in a humid atmosphere under 37 °C with 5% CO_2_. Trypsin/EDTA solution was used to culture these cells for the production of subcultures. The overexpression BNIP3 lentivirus and corresponding negative control were purchased from Genechem (Shanghai, China). The polybrene (Genechem) was used to promote the transfection efficiency. All the operations of the transfection were followed the instruction. 3-MA (5 mM/mL) (Sigma-Aldrich) was used to treat overexpression BNIP3 cells.

### Cell Counting Kit-8 (CCK-8) assay

ATDC5 cells were planted into the 96 well plates. After the adhesion of these cells, the culture medium supplemented with the LPS (0, 1, 2.5, 5, 10 μg/mL) was used for the culture of these cells for 6 h. Next, the CCK-8 solution (Dojindo, Kumamoto, Japan) was diluted with the culture medium (1:10) which then added into the 96 well plates. Then these cells were incubated in the incubator for 1.5 h. At last, the absorbance was measured with the spectrophotometer (Thermo Fisher Scientific).

### Detection of glycosaminoglycan (GAG)

The levels of total GAG were measured with the commercial kit from Jianglai Biotechnology Co., Ltd (Shanghai, China). Supernatant of ATDC5 cells was collected by centrifugal tube. Fifty microliters supernatant and 50 μL 1,9-dimethylmethylene blue (DMMB) were added to a 1.5-mL centrifuge tube, which was treated with vortex agitation for 15 s and incubated at room temperature for 30 min in dark. Then, the centrifuge tube was centrifuged at 16000 g for 10 min and the supernatant was removed cleanly. Fifty microliters propyl alcohol was added to the centrifuge tube for vortex agitation for 15 s and incubated at room temperature for 5 min in dark. The solution in the centrifuge tube was transferred to a new cuvette, which immediately detected by the spectrophotometer (Thermo Fisher Scientific). The relative levels of total GAG were determined according to the standard curve.

### ELISA assays

Supernatant of ATDC5 cells was collected by centrifugal tube. The supernatant was then centrifuged to remove impurities and transferred to sterilized tubes. Next, human TNF-α ELISA kit (ab181421, Abcam, UK), human IL-1β ELISA kit (RAB0273, Sigma Aldrich), and human IL-6 ELISA kit (ab178013, Abcam) were used to detect the secretion of tumor necrosis factor-α (TNF-α), interleukin-1 beta (IL-1β), and interleukin-6 (IL-6) of ATDC5 cells. All experimental operations were performed in accordance with the instructions.

### Apoptosis assays

Apoptosis kit (Beyotime, Nanjing, China) was used to determine the proportion of apoptosis cells. These cells were prepared into the cell suspension. Next, the cell suspension was washed with the cold phosphate buffer saline (PBS) for three times to eliminate the interference of fetal bovine serum. After that, these cells were incubated with the Annexin V and polyimide (PI) in the dark room for 40 min. At last, the flow cytometry was performed to detect the apoptosis rates of these cells.

### Western blotting

Total protein was collected with the RIPA buffer (Beyotime). And the concentration of these proteins was measured with the BCA (Beyotime) method. Next, these proteins were separated with the 10% sodium dodecyl sulfate polyacrylamide gel electrophoresis (SDS-PAGE) gel (Beyotime). After that, these proteins were transferred to the polyvinylidene fluoride (PVDF) membranes (Millipore, Massachusetts, USA). Then these membranes were blocked with the 5% skim milk powder and incubated with the primary antibodies at 4 °C overnight. The primary antibodies used in this research were BNIP3 (ab109362, Abcam), Bcl-2 (ab32124, Abcam), Bax (ab32503, Abcam), Cleaved caspase3 (ab49822, Abcam), Cleaved caspase9 (ab2324, Abcam), Little Computer 3 (LC3) I/II (ab62721, Abcam), Autophagy-related protein 7 (ATG7) (ab133528, Abcam), Beclin1 (#3495S, Cell Signaling Technology, USA), P62 (#5114S, Cell Signaling Technology), and GAPDH (ab8245, Abcam). On the second day, these membranes were washed with the phosphate-buffered solution (PBST) for three times and then incubated with the second antibody for 2 h at room temperature. At last, these membranes were washed with the PBST again and the immunoreactive signal was detected with the LAS-3000 Image Analyzer (Fujifilm, Tokyo, Japan).

### RT-PCR

Total mRNA was extracted with the trizol (Thermo Fisher Scientific) method. Next, the appropriate amount of mRNA was reversely transcribed into cDNA by the reverse transcription kit (Roche, Basle, Switzerland). Next, the SYBE Green (Applied Biosystems, New York, USA) was mixed with cDNA in a certain proportion. At last, those cDNA was amplified with the 7500 RT-PCR system (Applied Biosystems). The expression of targeted genes was calculated with the 2^−∆∆Ct^ method. The primers used in this research was BNIP3 forward: 5′-CAGAATTCATGGAGCAGAAACTCATCTCTGAAGAGGATCTGATGTCGCAGAACGGAGCG-3′ Reverse: 5′-TAGGATCCTCAAAAGGTGCTGGTGGAGG-3′ and GAPDH forward: 5′-ACAACTTTGGTATCGTGGAAGG-3′ Reverse: 5′-GCCATCACGCCACAGTTTC-3′.

### Immunofluorescence

These cells were plated on the sterilized coverslips before the experiment. After the adhesion of these cells, 4% paraformaldehyde was used to fix these cells. Next, the 0.2% Trixton-X 100 (Beyotime) was used to increase the penetrability of cell membranes. Then these cells were incubated with the primary antibody LC3B (#2775S, Cell Signaling Technology). Next, these cells were washed with the PBST for 30 min and incubated with the second antibody Alexa Fluor 555 Conjugate (#3969S, Cell Signaling Technology). After that, these cells were washed with the PBST again and the DAPI (Invitrogen, California, USA) was dropped on those cells. At last, these cells were observed under the laser scanning confocal microscope (Olympus, Hachioji, Japan).

### Statistical analysis

All the experiments in this research were repeated for three times. And the data in this paper was displayed as mean ± SD. All the data in this research was analyzed with the Graphpad Prism 7.0 (Graphpad Software Inc, California, USA). The difference between diverse groups was analyzed with the student’s *t* test. The difference was significant when the values of *P* was less than 0.05.

## Results

### The treatment with LPS leads to the downregulation of BNIP3 in chondrocytes

**Fig. 1:**
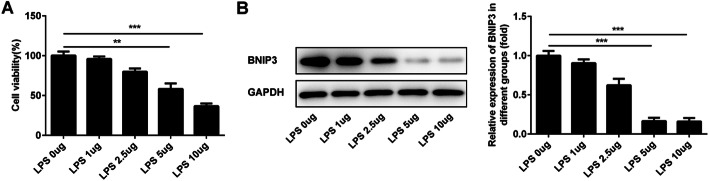
Treatment with LPS inhibited the expression of BNIP3 in chondrocytes. **a** The cell viability was determined with CCK-8 assays after the treatment with LPS. **b** The expression of BNIP3 in chondrocytes was determined with the western blotting after the treatment with LPS. *p < 0.05, **p < 0.01, ***p < 0.001

To clarify the expression of BNIP3 during the occurrence and development of osteoarthritis, we used the LPS to stimulate the ATDC5 cells and determined the levels of BNIP3 in these cells. As shown in Fig. 1a, the cell viability was gradually weakened after the treatment with LPS. After that, the expression of BNIP was detected with the western blotting. And, we found that BNIP3 is normally expressed in ATDC5 cells and BNIP3 expression was gradually decreased with the increasing dose of LPS (Fig. 1b). Given that the LPS (5 μg/mL) could maintain cell viability at the appropriate level and significantly inhibit the expression of BNIP3, we used the LPS (5 μg/mL) for the subsequent experiments.

### Overexpression of BNIP3 decreased the LPS-induced inflammation of chondrocytes

**Fig. 2:**
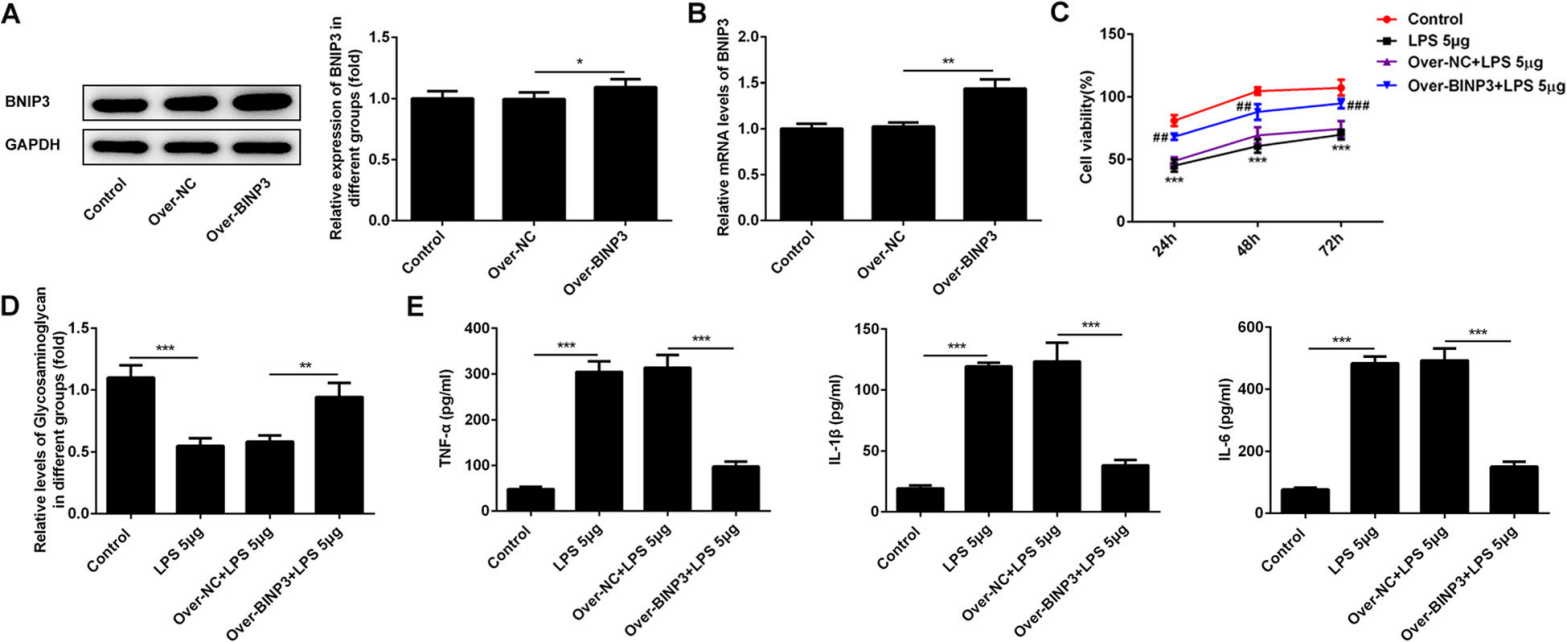
Overexpression of BNIP3 decreased the LPS-induced inflammatory injury of chondrocytes. **a**, **b** Western blotting and RT-PCR was performed to confirm the overexpression of BNIP3. **c** The cell viability of chondrocytes was detected with CCK-8 assays after the overexpression of BNIP3. **d** Levels of glycosaminoglycan in chondrocytes was determined with the kits after the overexpression of BNIP3. **e** The levels of IL-1β, IL-6, and TNF-α in the supernatant was determined with the ELISA assays after the overexpression of BNIP3. *p < 0.05, **p < 0.01, ***p < 0.001

For further research on the effect of BNIP3 on the development of osteoarthritis, we used the lentivirus to establish the overexpression BNIP3 ATDC5 cells. Next, the mRNA and protein levels of BNIP3 were detected with the RT-PCR and western blotting. The results (Fig. 2a and b) showed that the expression of BNIP3 was significantly elevated in these cells of the overexpression group. This result indicated that we have successfully constructed the overexpression BNIP3 chondrocytes. And, these cells could be used for the next experiments. After that, the CCK-8 assays were performed to detect the change of the cell viability after the overexpression of BNIP3. According to the results (Fig. 2c), we found that the overexpression of BNIP3 alleviated the LPS-induced damage for ATDC5 cells. The inflammatory response of chondrocytes is the critical trait of the osteoarthritis [20]. And, the GAG could play the anti-inflammatory role in diverse tissues [21]. Therefore, the levels of total GAG and pro-inflammation factors (TNF-α, IL-1β, and IL-6) were detected with the commercial kits. And the results (Fig. 2d) showed that the levels of total GAG were inhibited after the treatment with LPS. However, the production of glycosaminoglycan was recovered after the overexpression of BNIP3. Furthermore, the overexpression of BNIP3 abolished the LPS-induced TNF-α, IL-1β, and IL-6 in chondrocytes (Fig. 2e).

### Overexpression of BNIP3 decreased the LPS-induced apoptosis of chondrocytes

**Fig. 3:**
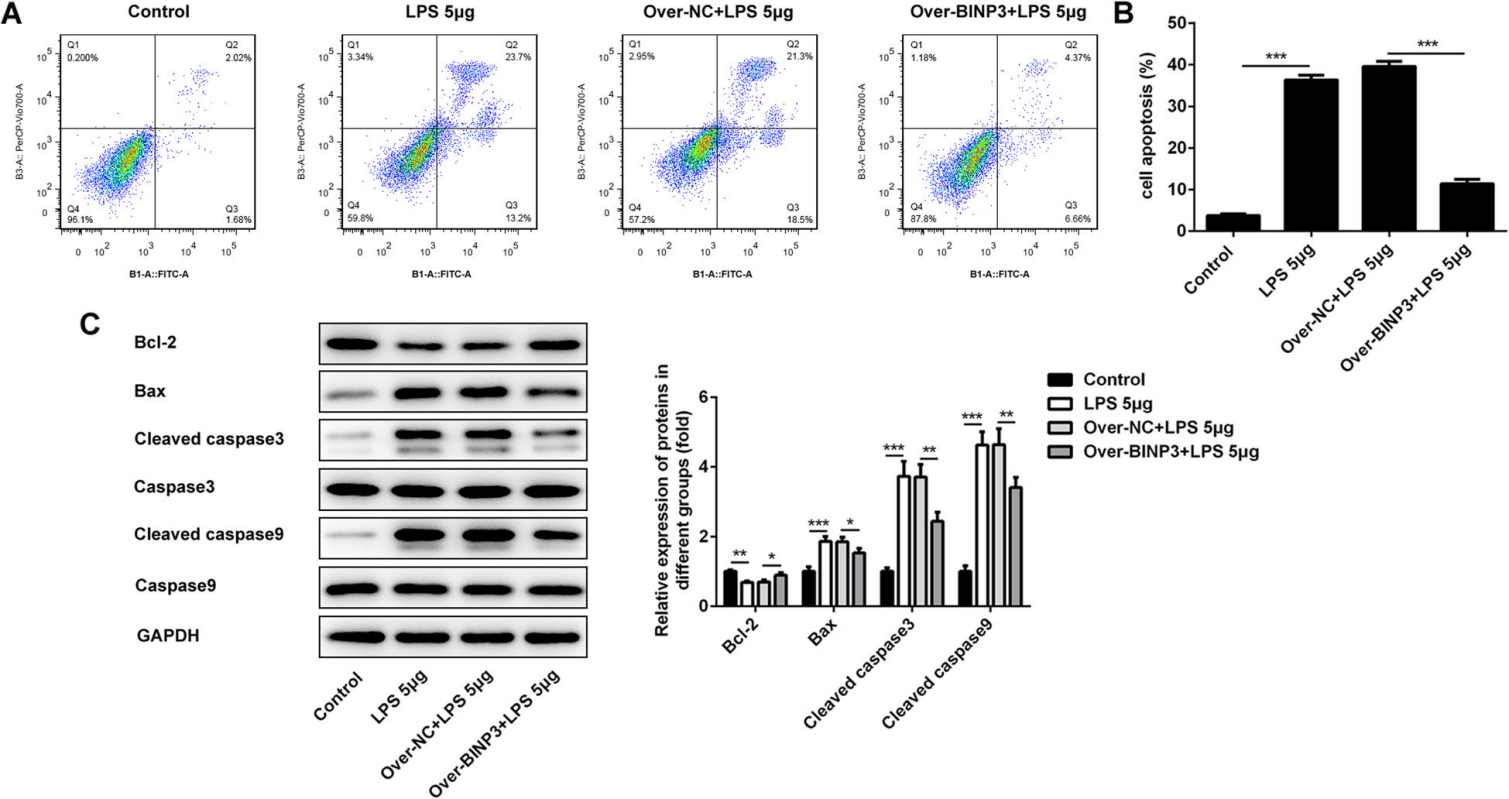
Overexpression of BNIP3 decreased the LPS-induced apoptosis of chondrocytes. **a**, **b** The apoptosis of chondrocytes was determined with the flow cytometry after the overexpression of BNIP3. **c** The expression of apoptosis-related proteins (Bcl-2, Bax, Caspase3, Cleaved caspase3, Caspase9, and Cleaved caspase9) was detected with the western blotting after the overexpression of BNIP3. *p < 0.05, **p < 0.01, ***p < 0.001

Cartilage destruction caused by chondrocyte apoptosis is a critical part of the occurrence and development of osteoarthritis [22]. Thus, we determined the proportion of apoptosis cells after the overexpression of BNIP3. As shown in Fig. 3a and b, the apoptosis rates of ATDC5 cells was enhanced after the treatment with LPS. Nevertheless, the overexpression of BNIP3 reduced the ratios of apoptosis cells. Next, the expression of apoptosis-related proteins was detected with the western blotting. And, we found that the levels of Bax, Cleaved caspase3, and Cleaved caspase9 were promoted after the treatment with LPS. Moreover, the expression of these proteins was renewedly inhibited after the overexpression of BNIP3. Furthermore, the expression of Bcl-2 was suppressed after the administration of LPS and rescued with the higher levels of BNIP3 (Fig. 3c).

### Overexpression of BNIP3 leads to the activation of autophagy in chondrocytes

**Fig. 4:**
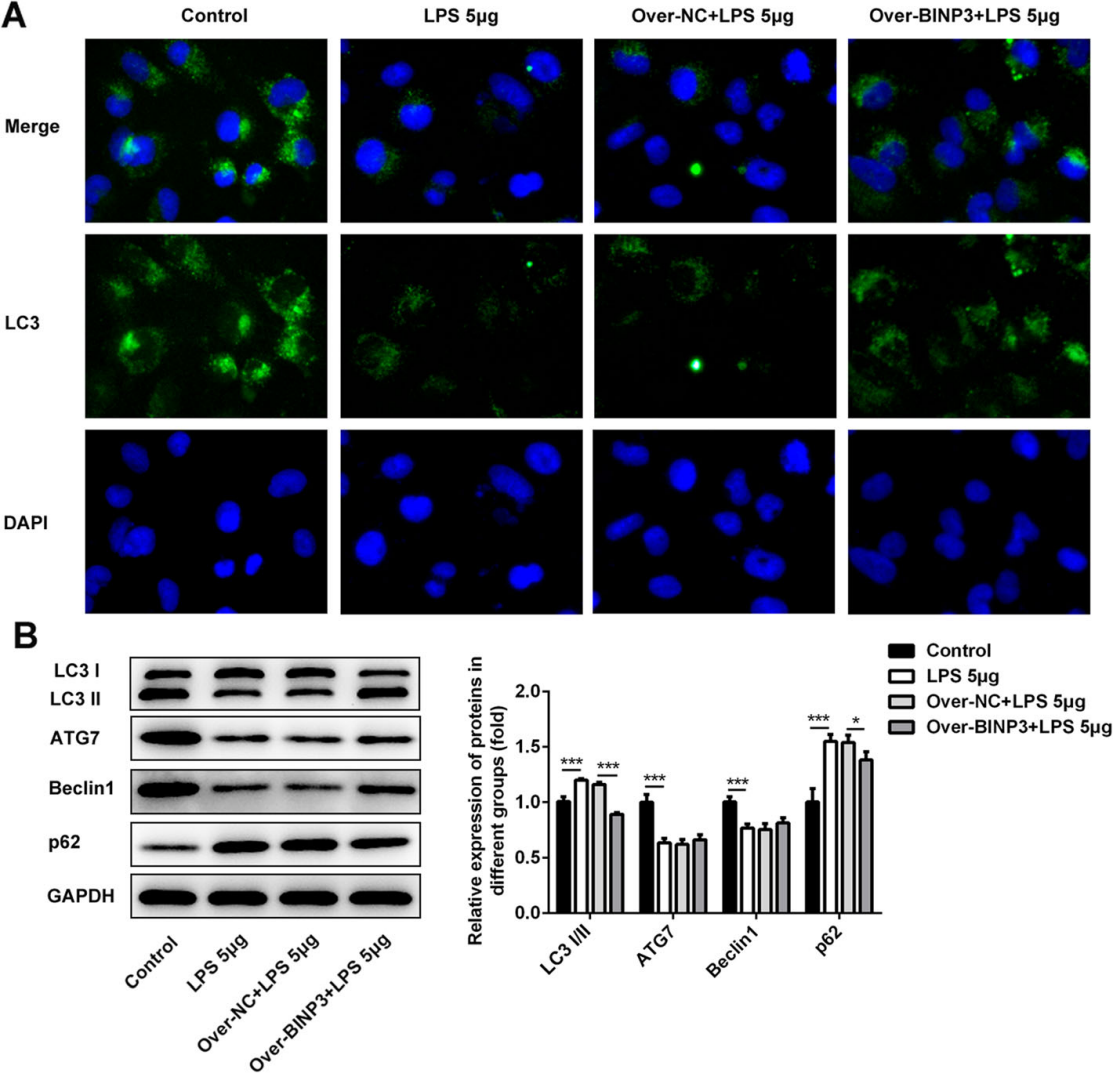
Overexpression of BNIP3 activated the autophagy of chondrocytes. **a** Representative images of immunofluorescence staining of LC3 in chondrocytes. **b** The expression of autophagy-related proteins (LC3I/II, ATG7, Beclin1, and P62) in chondrocytes was determined with the western blotting. *p < 0.05, **p < 0.01, ***p < 0.001

Previous study has shown that BNIP3 inhibited the expression of pro-inflammation factor by inducing the occurrence and development of autophagy [19]. In our study, we determined whether the BNIP3 could alleviate the LPS-induced inflammation and apoptosis by activating the autophagy. Therefore, the immunofluorescence was performed to reveal the alternation of expression of LC3 which is a biomarker of autophagy. As shown in Fig. 4a, the staining of LC3 was shallow in ATDC5 cells after the treatment with LPS. However, the staining recovered after the overexpression of BNIP3. Next, the levels of autophagy-related proteins were detected with the western blotting. From the results (Fig. 4b), we found that the expression of LC3I/II and P62 was enhanced while the levels of LC3I/II, ATG7, and Beclin1 were inhibited when the ATDC5 cells were stimulated with the LPS. After the overexpression of BNIP3, the levels of LC3I/II, ATG7, and Beclin1 were rescued while the expression of LC3I/II and P62 was suppressed.

### The effect of BNIP3 on the inflammation and apoptosis of chondrocytes was restricted after the inhibition of autophagy

**Fig. 5:**
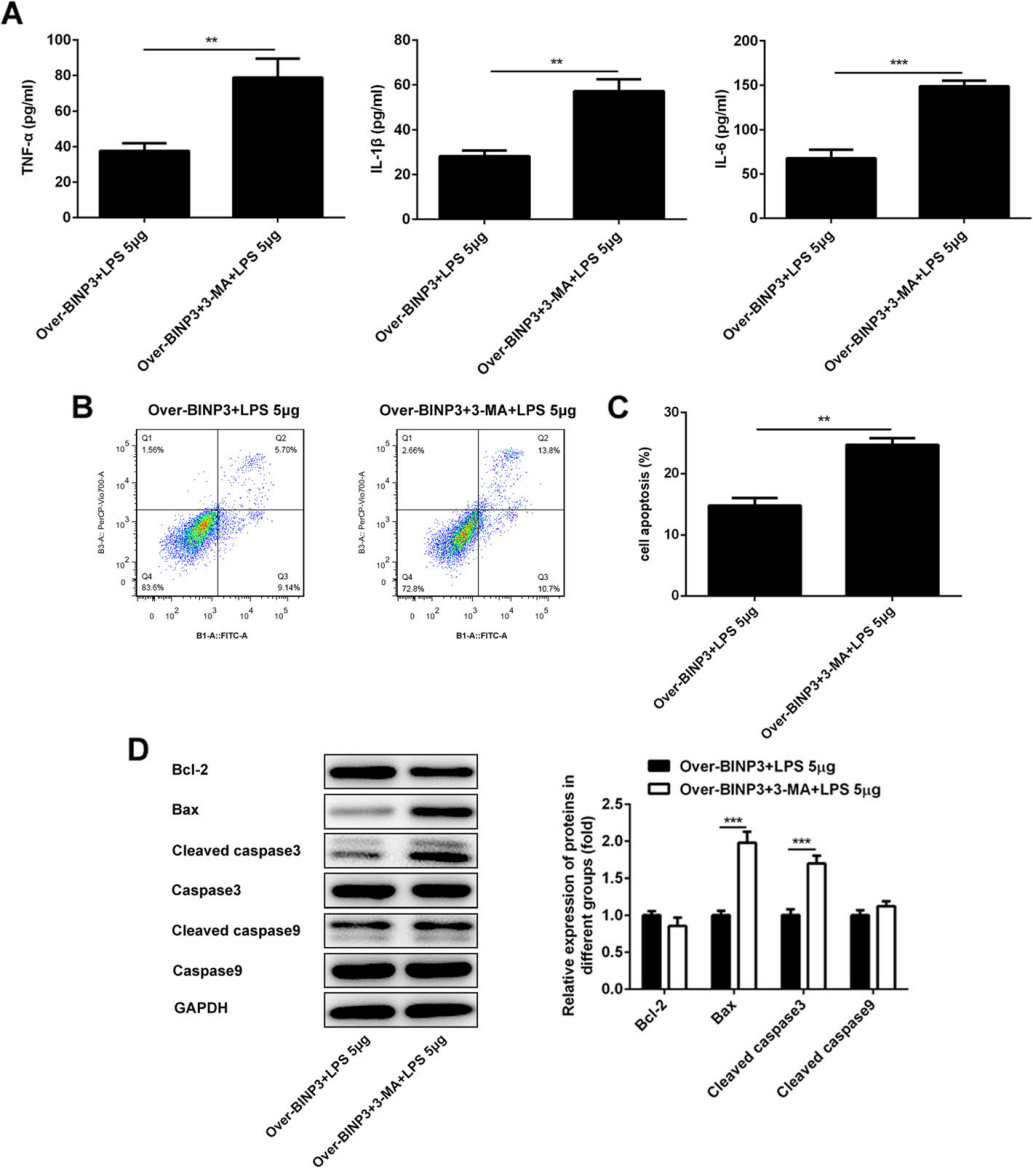
Suppression of autophagy weakened the inhibitory effect of BNIP3 on the inflammation injury and apoptosis of chondrocytes. **a** The levels of IL-1β, IL-6, and TNF-α in the supernatant of chondrocytes were determined with ELISA assays after the inhibition of autophagy. **b**, **c** The apoptosis rates of chondrocytes were detected with the flow cytometry after the suppression of autophagy. **d** Apoptosis-related proteins in chondrocytes was detected with the western blotting after the inhibition of autophagy. *p < 0.05, **p < 0.01, ***p < 0.001

In the last experiment, we used the 3-MA which is an inhibitor of autophagy to treat the overexpression BNIP3 ATDC5 cells. Next, the levels of TNF-α, IL-1β, and IL-6 were determined with the ELISA assays. According to the results (Fig. 5a), we found that the expression of TNF-α, IL-1β, and IL-6 was renewedly enhanced after the treatment of 3-MA. After that, the apoptosis rates of ATDC5 cells were detected with the flow cytometry. Result (Fig. 5b and c) showed that the administration of 3-MA promoted the proportion of apoptosis ATDC5 cells. The detection of apoptosis-related proteins further confirmed this result. As shown in Fig. 5d, the expression of Bax, Cleaved caspase3, and Cleaved caspase9 was increased while the levels of Bcl-2 were repressed after the treatment of 3-MA.

## Discussion

Osteoarthritis is one kind of the degenerative joint disease which induces severe joint pain and eventually causes disability [23]. Chondrocytes are the main cells in articular cartilage and play an important role in maintaining the normal physiological functions and morphology of cartilage. Studies generally believed that chondrocyte inflammation was a crucial link in the pathogenesis of osteoarthritis [24, 25]. Therefore, it is urgent to develop new treatments to alleviate the inflammation of chondrocytes and thus relieve the symptoms of osteoarthritis.

Furthermore, Autophagy is induced by multiple autophagic genes (light chain 3, Beclin-1, and other proteins). The occurrence of autophagy could maintain homeostasis of cells by removing damaged organelles, defective proteins, and external microorganisms (bacteria and viruses) [26, 27]. In essence, autophagy is a stress response of the cell, and this response enables the cell to survive under extreme conditions. And the molecular mechanism of autophagy is also related to most cellular stress response pathways [28]. And the inflammation and immunity-related pathways were also associated with the occurrence of autophagy [29]. The interaction between autophagy and immune or inflammation-related signaling pathways is complicated. Autophagy could induce and suppress the development of immune and inflammatory responses, and the changes of the expression of immune and inflammatory-related proteins could also affect the autophagy process. Furthermore, recent studies have found a relationship between the occurrence of autophagy and the development of inflammatory responses in many tissues [30, 31]. There is a study suggesting that the progranulin suppresses the inflammatory response by promoting the development of autophagy [32]. In addition, the autophagy also revealed that could induce the inactivation of inflammasome and relieve the skin inflammation [33].

Previous study has proved that the BNIP3 can induce the occurrence and development of autophagy [34]. Furthermore, there is also a study revealing that higher levels of BNIP3 suppress the secretion of IL-18 in retinal pigment epithelium cells [19]. And in our study, we found that the expression of BNIP3 was inhibited after the treatment with LPS. The treatment with LPS led to the inflammation of chondrocytes. However, the overexpression of BNIP3 decreased the inflammation of these cells. Light chain 3 (LC3I/II) protein is a biomarker of the autophagy. During the process of autophagy, LC3I/II could be converted to LC3I/II by enzymatic hydrolysis [27]. In addition, the higher levels of ATG7 and Beclin-1 also played a critical role during the autophagy process [35]. And in eukaryotic cells, p62 could promote the degradation of ubiquitinated proteins in autophagosomes by directly binding to these proteins [36]. In this study, we found that the expression of LC3I/II, ATG7, and Beclin-1 was inhibited and the levels of LC3I/II and P62 were enhanced after the treatment with LPS. Moreover, the overexpression of BNIP3 abolished the changes of the expression of these proteins. And the secretion of proinflammatory factors (TNF-α, IL-1β, and IL-6) was rescued when the autophagy process was suppressed by the autophagy inhibitor (3-MA). These results indicated that BNIP3 decreased the LPS-induced inflammatory damage of chondrocytes by activating the autophagy process.

On the other hand, the apoptosis of chondrocytes is also one of the pathogenic mechanisms of osteoarthritis [37, 38]. There is a study also revealing that the autophagy relieves the apoptosis of cells during the intestinal ischemia-reperfusion [39]. In our study, the treatment with LPS induced the apoptosis of chondrocytes. However, the apoptosis rates of these cells were declined after the overexpression of BNIP3. After the application of 3-MA, the proportion of apoptosis cells was recovered. These results also suggested that BNIP3 decreased the apoptosis of these cells by inducing the occurrence of autophagy.

## Conclusion

Overall, we detected the effect of BNIP3 on the inflammation and apoptosis of chondrocytes in this research. And our results revealed that the BNIP3 alleviated the LPS-induced inflammation and apoptosis of chondrocytes by activating the autophagy process. Our study also provided the potential target and therapy for the clinical treatment of osteoarthritis.

## Data Availability

The datasets generated/analyzed during the current study are available.

## Abbreviations

BNIP3: BCL2/adenovirus E1B 19 kDa protein-interacting protein 3
LPS: Lipopolysaccharide
ELISA: Enzyme-linked immunosorbent assay
TNF-α: Tumor necrosis factor-α
IL-1β: Interleukin-1 beta
IL-6: Interleukin-6
LC3: Little computer 3
ATG7: Autophagy-related protein 7
DMEM: Dulbecco’s Modified Eagle Medium
GAG: Glycosaminoglycan
PBS: Phosphate buffer saline
PI: Polyimide
SDS-PAGE: Sodium dodecyl sulfate-polyacrylamide gel electrophoresis
PVDF: Polyvinylidene fluoride
PBST: Phosphate-buffered solution
DMMB: 1,9-dimethylmethylene blue
